# Microbial Technologies Enhanced by Artificial Intelligence for Healthcare Applications

**DOI:** 10.1111/1751-7915.70131

**Published:** 2025-03-18

**Authors:** Taeho Yu, Minjee Chae, Ziling Wang, Gahyeon Ryu, Gi Bae Kim, Sang Yup Lee

**Affiliations:** ^1^ Metabolic and Biomolecular Engineering National Research Laboratory, Department of Chemical and Biomolecular Engineering (BK21 Four) KAIST Institute for BioCentury, Korea Advanced Institute of Science and Technology (KAIST) Daejeon Republic of Korea; ^2^ Systems Metabolic Engineering and Systems Healthcare Cross‐Generation Collaborative Laboratory KAIST Daejeon Republic of Korea; ^3^ Graduate School of Engineering Biology KAIST Daejeon Republic of Korea; ^4^ BioProcess Engineering Research Center KAIST Daejeon Republic of Korea; ^5^ Center for Synthetic Biology KAIST Daejeon Republic of Korea

**Keywords:** artificial intelligence, drug, healthcare, microbial technology

## Abstract

The combination of artificial intelligence (AI) with microbial technology marks the start of a major transformation, improving applications throughout biotechnology, especially in healthcare. With the capability of AI to process vast amounts of biological big data, advanced microbial technology allows for a comprehensive understanding of complex biological systems, advancing disease diagnosis, treatment and the development of microbial therapeutics. This mini review explores the impact of AI‐integrated microbial technologies in healthcare, highlighting advancements in microbial biomarker‐based diagnosis, the development of microbial therapeutics and the microbial production of therapeutic compounds. This exploration promises significant improvements in the design and implementation of health‐related solutions, steering a new era in biotechnological applications.

## Graphical abstract

Microbial technologies combined with artificial intelligence can enhance healthcare by predicting microbiome biomarkers for disease diagnosis, developing microbial therapeutics, discovering and designing drugs and producing therapeutic compounds through microbial processes.

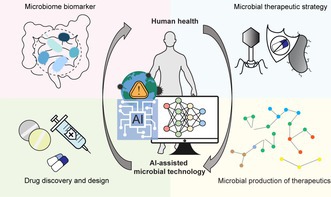

## 1 Introduction

Throughout the history of technological advancements, biotechnologies have consistently demonstrated their usefulness, evolving from traditional practices such as fermentation for food production to recent efforts to combat diseases. Microbial technology, an essential branch of biotechnology, harnesses whole microorganisms or their cellular components, including bacteria, archaea, fungi, algae and viruses. The advent of artificial intelligence (AI) has initiated a fundamental shift in microbial technology, revolutionising its applications across all fields of biotechnology. Moreover, having witnessed AI's capabilities to accelerate drug discovery and design during the COVID‐19 pandemic, the integration of microbial technology with AI has become indispensable for the healthcare industry (Zhou et al. 2020). AI, a machine (or software) capable of simulating aspects of perception and reasoning through advanced data processing and learning algorithms, has been used to solve challenges beyond the scope of conventional methods. Particularly, the vast accumulation of biomedical data and advancements in state‐of‐the‐art deep learning models have enabled AI to understand complex biological systems through data‐driven approaches, often without prior knowledge of these systems. In this context, AI in microbial technologies has emerged as a pivotal breakthrough in all aspects of healthcare, ranging from disease diagnosis to treatment (Durant et al. 2024; Zhang et al. 2025). In this review, we discuss the applications of advanced microbial technologies integrated with AI in healthcare, elucidating how microbial technology promotes health monitoring and diagnosis, facilitates the development of microbial therapeutics and enables the design and production of drugs and natural compounds relevant to improved health.

## 2 Health Monitoring and Diagnosis Using AI‐Assisted Microbial Biomarkers

In modern times, global health threats are increasingly associated with microbes, especially pathogens. Notably, half of the top ten health threats announced by the World Health Organisation (WHO) in 2019 are related to viruses (influenza, Ebola, dengue and human immunodeficiency virus) or antimicrobial‐resistant bacteria. As pathogens and viruses continuously evolve to evade conventional public health measures, it becomes crucial to trace and forecast these mutations to prepare public health systems (Meckawy et al. 2022). However, the challenge of predicting and detecting important mutations often surpasses human capabilities.

**FIGURE 1:**
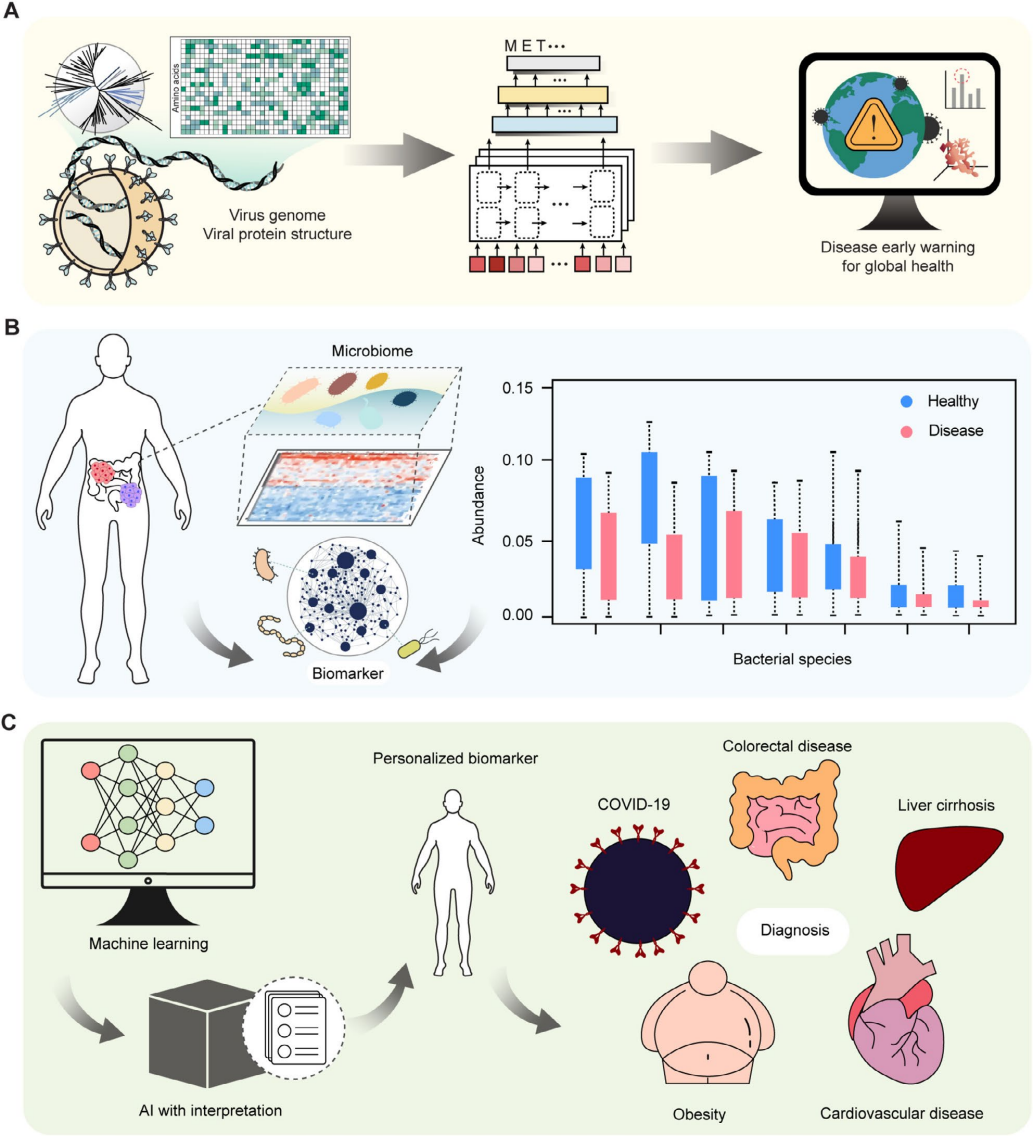
AI‐assisted microbial biomarker for health monitoring and diagnosis. (A) AI for the prediction of immune‐evading variants. (B) The discovery of microbiome‐based biomarkers at the bacterial species level. (C) The interpretation of the AI reasoning process leads to the discovery of personalised biomarkers with the advancements in AI.

In this regard, AI technology proves to be invaluable in predicting immune‐escaping variants, which conventional methods struggle to detect (Figure 1A). For instance, a protein language model, a language model trained on large‐scale protein sequences to learn representations associated with protein function and structure, has been employed to identify potential immune‐escaping variants (Beguir et al. 2023). This model, pre‐trained on 270 million protein sequences from UniRef100, which is a large collection of diverse protein sequences, was fine‐tuned for the spike proteins of SARS‐CoV‐2 from the Global Initiative on Sharing All Influenza Data (GISAID) to predict the ability of variants to escape the immune system and their propensity to proliferate. By combining a protein structure‐based binding affinity and epitope analysis module, the system was able to predict a list of high‐risk variants that would arise, providing a monitoring and risk‐evaluating system for early pandemic warnings. Another AI tool, EVEscape, incorporates three modules. The first module employs a Bayesian variational autoencoder to encode viral sequences into a latent space, predicting the probability of the viral fitness changes on mutations (Thadani et al. 2023). The other two modules analyse protein structures to assess antibody accessibility and dissimilarity. EVEscape was trained on viral sequence data available before January 2020, including coronavirus sequences. Despite being trained on the pre‐pandemic data, the top 50% of predicted receptor‐binding domains of SARS‐CoV‐2 from GISAID had been observed during the pandemic by May 2023. EVEscape not only predicts newly emerging SARS‐CoV‐2 strains with immune‐escaping capabilities based on previously reported data, but also has the generalisation ability to identify circulating strains of other viral families, including Lassa virus, Nipah virus and HIV. These predictive abilities of AI, coupled with microbial data, including genomic features that infect humans, enable the early identification of potential high‐risk variants, thereby facilitating the proactive development of vaccines and therapeutics and guiding strategic public health planning to mitigate emerging outbreaks (Mollentze et al. 2021; Saha et al. 2021).

The microbiome has been linked to a variety of diseases, suggesting that the human microbiome holds potential as a biomarker for distinguishing patients from healthy individuals (Figure 1B) (Sekirov et al. 2010; Segata et al. 2011; Hou et al. 2022). Along with 16S ribosomal RNA sequencing, which has been widely employed to profile microbial communities, metagenomics has been used to investigate the diversity and dysbiosis of the intestinal microbiome and its correlation with disease (Wang et al. 2015; Knight et al. 2018). Recent developments in machine learning, coupled with the abundance of microbiome data, facilitate the exploration of intricate correlations where various diseases share common microbiome signals (Goodswen et al. 2021). For example, a support vector machine (SVM), a machine learning algorithm that finds the optimal hyperplane to maximise the margin between classes, was trained on gut microbiome data that were collected from 181 individuals diagnosed with liver cirrhosis (Qin et al. 2014). Employing a pattern recognition technique and the minimum redundancy–maximum relevance method, 15 biomarkers were identified that distinguished patients based on their gut microbiota profiles. Subsequently, SVMs were utilised to accurately identify patients from healthy individuals, achieving the area under the receiver operating characteristic curve (AUROC) of 0.838, a metric that assesses classification performance by comparing the true positive and false positive rates across various decision thresholds. Another study reported the development of machine learning models capable of predicting eight types of diseases (i.e., colorectal cancer, colorectal adenomas, Crohn's disease, ulcerative colitis, irritable bowel syndrome, obesity, cardiovascular disease and post‐acute COVID‐19 syndrome) from faecal metagenomics data (Su et al. 2022). This approach aimed to discern disease‐specific microbial compositions, despite the presence of common microbial signatures across diseases. The study trained five machine learning models on profiles of faecal microbiome at the species level: SVM, k‐nearest neighbours, which is a machine learning algorithm that predicts outcomes by analysing the k nearest data points in the feature space; random forest, which is an ensemble machine learning algorithm that builds multiple decision trees during training and combines their predictions; multilayer perceptron, which is a machine learning algorithm consisting of fully connected layers with nonlinear activation functions for learning complex patterns; and graph neural network, which is a deep learning model designed to process graph‐structured data by learning node, edge, or graph representations. The microbiome data were collected from 2320 individuals to distinguish between nine phenotypes that include eight disease types and a healthy control. The machine learning models achieved the AUROC from 0.67 to 0.90 across the nine phenotypes in the test dataset. The high performance of the AI suggests the possibility of utilising noninvasive methods for diagnosing significant human diseases.

While machine learning has been widely employed for disease classification using microbiome data, its clinical application remains limited due to the lack of interpretability in AI's complex reasoning process. AI models often generate predictions without clearly indicating which features contributed to the decision. This lack of transparency makes it difficult for clinicians and researchers to evaluate the reliability of the model. To address this limitation, recent studies have explored approaches to interpret AI reasoning processes. In a recent study, Shapley Additive Explanations (SHAP), a local explanation technique that calculates the importance of each input feature for the prediction, was employed to provide an interpretation of the reasoning process of AI for the diagnosis of colorectal cancer (CRC) (Figure 1C) (Rynazal et al. 2023). A random forest model was trained with bacterial species abundance data of the gut microbiome to distinguish between CRC patients and healthy individuals. SHAP enabled the discovery of personalised biomarkers that contribute to the diagnosis of CRC at the bacteria species level. For example, *Fusobacterium nucleatum* , known as a representative microbe related to CRC, contributed a SHAP value of 0.05 in one patient, which accounts for 25% of the increase from the average prediction score (0.53) to the final prediction score (0.73) for the patient. In contrast, it did not affect the prediction for another CRC patient, implying the importance of AI with interpretation for the development of precision medicine.

Advances in graph neural networks have enabled the analysis of biomarkers based on microbial interactions, rather than just species abundance. Weighted signed graph convolutional neural network for microbial biomarker identification (WSGMB), an AI model utilising microbial co‐occurrence networks, was developed with graph neural networks (Pan et al. 2024). These neural networks were trained to predict healthy states from microbial co‐occurrence networks and identified disease‐related biomarkers by assessing changes in prediction scores when microbial nodes were perturbed. This network‐based approach achieved the AUROC exceeding 0.7 for predicting known CRC‐related bacteria.

In contemporary society, global health threats are increasingly linked to viruses and antimicrobial‐resistant bacteria. Addressing these challenges, the importance of microbial biomarkers facilitated by AI technology is becoming prominent. Machine learning techniques based on microbial data offer innovative opportunities for diagnosing and predicting various diseases. However, the utilisation of such technology necessitates overcoming challenges such as interpretation difficulties and ensuring data accuracy. Consequently, the integration of AI with microbial data holds promise for enabling more precise disease diagnosis in the future. Such advancements are expected to play an important role in establishing more efficient and accurate public health systems.

## 3 Therapeutic Strategies Utilising AI and Microbes

The human microbiome is recognised as another organ of the human body due to its importance, and managing it holds significant potential for improving health conditions (Baquero and Nombela 2012). Therefore, maintaining a healthy microbiome is acknowledged as a novel class of therapeutics to prevent and treat diseases by addressing dysbiosis through interventions such as probiotics or faecal microbiota transplantation (Pamer 2014; Sorbara and Pamer 2022). For example, the faecal microbiota biotherapeutic Rebyota was first approved by the FDA in 2022 (Walter and Shanahan 2023), and the oral microbiota biotherapeutic Vowst received FDA approval in 2023 (Allegretti et al. 2023), both preventing recurrent *Clostridioides difficile* (formerly *Clostridium difficile* ) infection.

**FIGURE 2:**
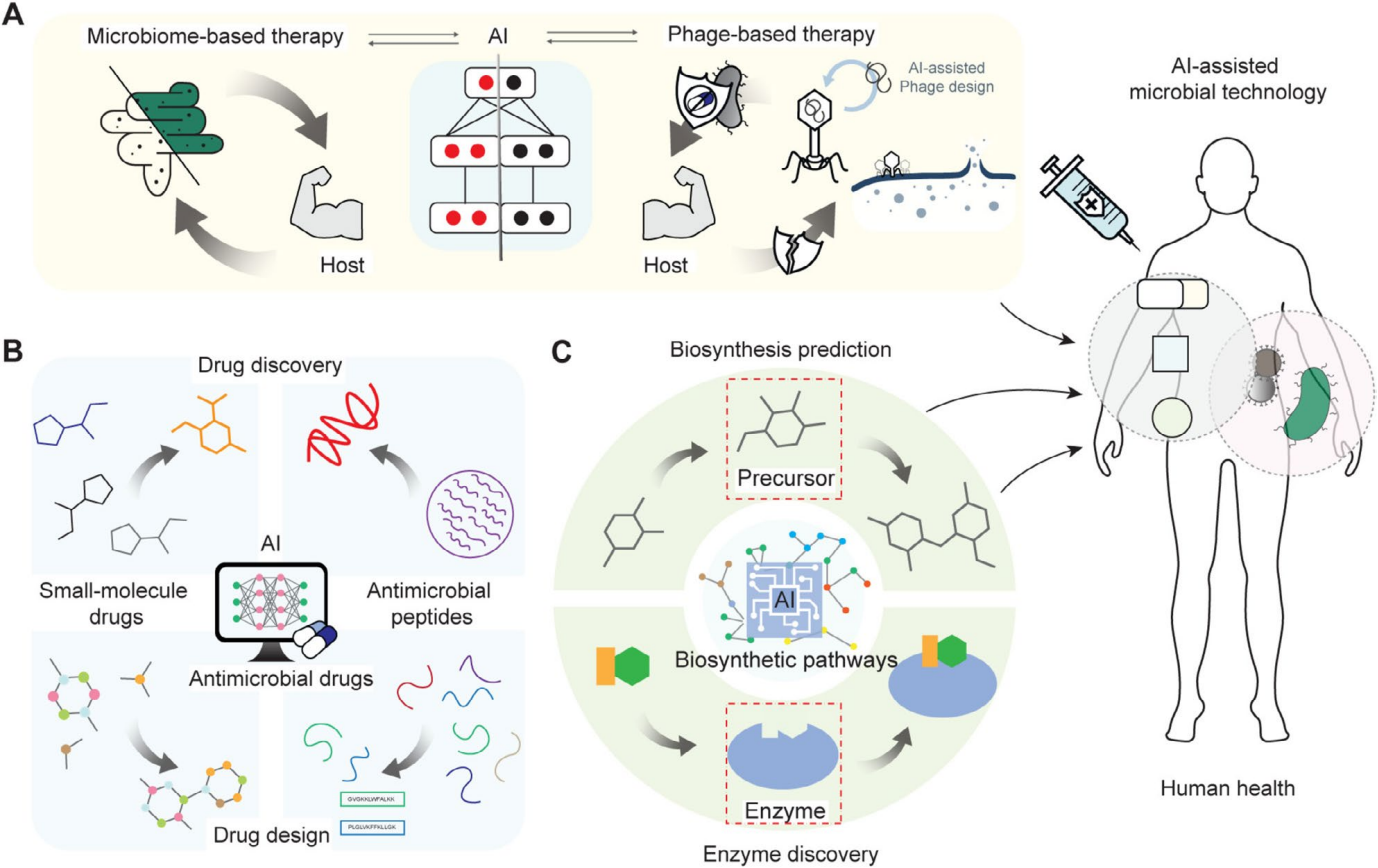
Application of AI‐assisted microbial technology in human health. (A) Microbiome‐based therapy and phage‐based therapy leverage AI to enhance understanding of microbial interactions, facilitating personalised treatments for maintaining a healthy microbiome and targeting bacterial infections. (B) AI accelerates the discovery of small‐molecule antimicrobial drugs and peptides with low resistance potential, while also contributing to the design of potent molecules and peptides, revolutionising microbial drug discovery and design. (C) AI‐driven biosynthesis prediction and enzyme discovery in metabolic engineering enable sustainable production of therapeutic chemicals, offering diverse microbial‐produced therapeutic options for societal health and sustainability.

To harness the microbiome as a therapeutic, it should be controlled to minimise unintended interactions among microorganisms or between the microbiome and hosts. This approach necessitates a comprehensive understanding of the associations between the microbiome and hosts. Recent applications of AI have contributed to the understanding of these complex associations. For example, a deep learning model named mNODE was developed by using neural ordinary differential equations, which integrate neural networks with ordinary differential equation solvers (Wang et al. 2023). The model was trained on microbial composition and metabolomic profiles from the PRISM dataset, comprising 68 Crohn's disease patients, 53 ulcerative colitis patients and 34 non‐inflammatory bowel disease (non‐IBD) controls, to predict metabolome profiles from microbial data. By quantifying the impact of changes in specific microbial abundance on metabolite concentrations, the AI provided insights into microbe–metabolite interactions with potential applications in precision nutrition and microbiome‐based interventions. Host–microbe interactions have also been analysed through advances in AI (Figure 2A). A recent study reported the development of a machine learning framework, which integrated sparse canonical correlation analysis, a dimensionality reduction technique that identifies correlations between two high‐dimensional datasets while selecting the most relevant features, and lasso regression, a regularisation method that uses an L1 penalty, to investigate associations between gut microbes and host genes (Priya et al. 2022). The framework was trained on 208 pairs of gut microbiome profiles and host transcriptome data from colonic mucosal samples of CRC patients and IBD patients, using gut microbiome abundance and host gene expression levels as inputs to predict disease‐specific patterns in microbiome–host gene associations. It revealed that while patients with different diseases may share similar microbiomes, the associations between the microbiome and host genes can vary.

Microbial technology with AI has also advanced the treatment of bacterial infections with phage therapy, which utilises bacteriophages as therapeutics against pathogenic bacterial infections (Chan et al. 2013; Lin et al. 2017). Due to its high specificity for host strains and effectiveness against antibiotic‐resistant bacteria, phage therapy has attracted more attention as an alternative to antibiotic treatment in recent years. Phage therapy has been performed as a phage cocktail, which is a mixture of various bacteriophages to target a broad range of hosts and to lower the chance of antimicrobial resistance. However, the complex nature of the cocktail, such as interactions between phages, stability and safety of the phages, has limited the application of bacteriophages in clinical settings. AI improves the accessibility of phage therapy by predicting phage–host interactions, thereby providing possible mechanisms of phage infection to guide the development of phage therapy. DeepHost, a machine learning model, was developed using convolutional neural networks (CNN) that can effectively capture spatial patterns in input data to identify the host species of a phage by analysing its genomic data (Ruohan et al. 2022). The model was trained on a dataset comprising 7483 phage genomes and 118 host genomes, achieving 90.78% accuracy in predicting the host of given phages at the species level. CL4PHI, a contrastive learning‐based AI, has also been developed for the prediction of phage–host relationships without being limited to predetermined host types, where contrastive learning is a self‐supervised approach that trains the model to distinguish similar and dissimilar pairs (Zhang, et al. 2023a). To achieve this, the model was trained to bring the representation of paired genome sequences (i.e., a phage and its corresponding host genome sequences) close together in the latent space, using two benchmark datasets, the DeepHost dataset, which includes 8756 phage genomes with 118 bacterial host species, and the CHERRY dataset, which includes 1940 phages with 223 bacterial hosts from CHERRY. This approach enabled host prediction for target phages by measuring the distances between their learned representations. Additionally, integrating protein structural information with sequence‐based approaches enhances the ability to capture host specificity. For predicting bacteriophage hosts, PHIStruct, a multilayer perceptron model, was trained on a dataset of 7627 receptor‐binding protein structures from 3350 phages by employing SaProt embeddings (Su et al. 2023; Gonzales et al. 2025). These embeddings extract learned representations of proteins from a protein language model pre‐trained on both protein sequences and structures. PHIStruct outperformed models using either sequence or structural information alone, demonstrating that incorporating structural data improves the prediction of novel phage sequences with low similarity to known phages.

Beyond the classification and regression of microbial characteristics, generative AI now plays an essential role in the development of phage therapy. To address the deficiency of phage–host interaction data, which is required for the training of deep learning models, a recent study developed PHIAF, a deep learning model trained on a dataset consisting of 5399 phage–host interactions between 5331 phages and 235 bacterial hosts (Li and Zhang 2022). To enhance model performance, PHIAF employs a generative AI, a technique that learns data distributions to generate synthetic samples that resemble the actual data, thereby generating pseudo‐phage–host interactions for data augmentation. Such advances in AI for phage–host interaction are expected to facilitate more precise treatments for bacterial diseases, enabling the expansion of phage therapy into personalised prescription and rational design. These evolving tools hold potential to advance microbial therapeutic fields, including microbiome‐based therapies.

## 4 Design and Production of Antibiotics Using AI‐Assisted Microbial Technology

In recent years, the emergence of antibiotic‐resistant strains has underscored the urgent need for novel antimicrobial drugs. The traditional drug discovery and development pipeline for antimicrobials, characterised by its lengthy timeline and substantial costs, has prompted a growing interest in computational methodologies capable of accelerating the discovery of drug candidates. The development of AI has paved the way for AI‐assisted microbial technologies to facilitate the discovery of small‐molecule antimicrobial drugs (Figure 2B) (Sadybekov and Katritch 2023).

One application of AI, particularly graph neural networks, has enabled the discovery of promising small‐molecule antimicrobial compounds (Liu et al. 2023). Using an in‐house dataset of 480 active and 7204 inactive molecules, neural networks were trained for binary classification to predict molecules likely to exhibit antibiotic activity against *Acinetobacter baumannii* . The trained neural networks were used to screen Drug Repurposing Hub, a library of 6680 structurally diverse chemicals, leading to the discovery of a novel antimicrobial compound, abaucin. Abaucin belongs to a novel structural class, distinct from existing antibiotic classes, which are limited in their long‐term clinical applicability due to the high prevalence of antibiotic resistance. Furthermore, AI has extended its reach beyond small molecules to aid in the discovery of antimicrobial peptides, a class of small proteins garnering increasing attention due to their relatively low likelihood of inducing antimicrobial resistance (Stokes et al. 2020; Melo et al. 2021). For antimicrobial peptide discovery, a pipeline including long short‐term memory (LSTM) which is a type of recurrent neural network that uses gate mechanisms to effectively learn long‐range dependencies in sequential data, attention‐based networks, which assign weights to different regions of the input sequence to focus on the most relevant regions, and bidirectional encoder representation from transformers (BERT) which is a language model that leverages attention networks and bidirectional context to learn representations of sequences, was developed, and remarkable success has been achieved in screening putative peptides to identify novel peptides (Ma et al. 2022). The AI‐assisted pipeline not only demonstrated a high positive rate (83.8%) for antimicrobial peptide discovery but also enabled the discovery of novel peptides with low sequence similarity to those in the training dataset, indicating potential for novel functional and structural diversity.

Moreover, the advent of generative AI has revolutionised the design of antimicrobial drugs. In an effort to design small‐molecule drugs, SyntheMol was developed to generate molecules possessing antibiotic activity against *A. baumannii* by using antibiotic activity prediction models combined with a Monte Carlo tree search, exploring decision trees by simulating numerous possible paths and refining choices based on probabilistic outcomes (Swanson et al. 2024). The Monte Carlo tree search explored combinations of molecular building blocks derived from a dataset of 13,524 molecules, which include 470 bioactive compounds screened against *A. baumannii* to design molecules exhibiting potent antibiotic activity, guided by antibiotic activity prediction models that employ graph neural networks and random forests. The sequential exploration of building block combinations enabled not only the discovery of novel drugs but also the identification of feasible synthetic pathways for these drugs. Another example of AI in small‐molecule drug design is TamGen, a GPT‐like generative AI that autonomously generates simplified molecular input line entry system (SMILES) strings of drug candidates (Wu et al. 2024). TamGen integrates a transformer‐based protein encoder that captures both sequential and structural properties of target proteins, a variational autoencoder‐based compound encoder that optimises compound generation, and a GPT‐like chemical language decoder for drug design. This architecture enables the encoding of target protein information and query compound details, facilitating the creation of novel drugs tailored to specific target proteins. By pre‐training on 10 million SMILES strings from PubChem and fine‐tuning on a dataset of 100,000 drug‐target pairs from CrossDocked2020, TamGen successfully designed 14 compounds capable of inhibiting Caseinolytic protease P of *Mycobacterium tuberculosis* . In another study for antimicrobial peptide design, HydrAMP was trained to generate antimicrobial peptides with desired activities by employing a conditional variational autoencoder, which learns a low‐dimensional latent space while incorporating conditions, enabling controlled generation of new samples with predefined properties (Szymczak et al. 2023). The model was trained on a curated dataset of peptide sequences of up to 25 amino acids, including known antimicrobial peptides, peptides with reported minimal inhibitory concentration values and nonredundant sequences from UniProt. HydrAMP was optimised for both unconstrained and analogue peptide generation, allowing it to produce novel antimicrobial peptides and modify inactive peptides into bioactive forms. The research demonstrates the application of generative AI in designing novel small‐molecule antimicrobial drugs as well as antimicrobial peptides structurally. Additionally, AI interpretation aids in designing peptides with enhanced antimicrobial activity. EvoGradient was developed to identify critical amino acids influencing antimicrobial activity predictions, which are subsequently targeted for mutation (Wang et al. 2025). To achieve this, the model was trained using a dataset of 24,766 antimicrobial peptides and 26,047 nonantimicrobial peptides, enabling it to distinguish key sequence features associated with antimicrobial activity. By analysing gradients from backpropagation in antimicrobial activity prediction models, EvoGradient guides the evolution of peptides, pinpointing mutations that enhance antimicrobial activity. This approach extends AI's role from identifying potential drugs to optimising their effectiveness. In summary, the development and application of AI have brought new opportunities and challenges in the discovery and development of drugs for microbial‐related diseases.

The discovered or designed therapeutic chemicals can be produced via chemical synthesis, or in the case of phytochemicals, through extraction from plants. However, drawbacks such as low yields, the use of toxic chemicals, or extensive land requirements for harvesting necessitate alternative production methods. Recent advances in metabolic engineering enable the sustainable production of complex therapeutic chemicals (Choi et al. 2019; Lee et al. 2019; Jang et al. 2022; Yang et al. 2022; Zhou et al. 2024), including anticancer drugs (Ajikumar et al. 2010; Zhang et al. 2022), tropane alkaloids for neuromuscular disorders (Srinivasan and Smolke 2020) and opioids for pain management (Galanie et al. 2015), through the development of microbial cell factories (Choi et al. 2019; Lee et al. 2019; Jang et al. 2022; Yang et al. 2022; Zhou et al. 2024). Genome‐scale metabolic simulation (Gu et al. 2019) has become an essential tool to efficiently develop microbial strains capable of producing these chemicals. Despite successful demonstrations of microbial production, challenges persist in broadening the bio‐based production portfolio. AI can facilitate the development of microbial cell factories for the production of therapeutic chemicals (Figure 2C) (Yuan et al. 2023). For example, AI‐driven prediction of biosynthetic pathways allows for the metabolic pathway reconstruction of natural products with complex structures. RDEnzyme, employing 6973 reaction rules, predicts precursors for a target chemical, filtering out biotransformations that require complex precursors or unavailable enzymes using multilayer perceptrons (Sankaranarayanan et al. 2022). The recursive application of the reaction rules and AI‐based filters enabled the prediction of multi‐step biosynthetic pathways for antiviral drugs, Islatravir and Molnupiravir.

Recent advances in machine translation also inspired researchers to perform retrobiosynthesis by translating a target chemical into a precursor, without the use of reaction rules. BioNavi‐NP, a representative retrobiosynthesis tool without reaction rules, integrates transformer networks that perform single‐step retrobiosynthesis with the A* search algorithm, a heuristic graph search algorithm that prioritises the most promising pathway expansions to efficiently explore routes (Zheng et al. 2022; Zeng et al. 2024). Being trained on 33,710 biosynthetic reactions from BioChem and 62,370 organic reactions from the USPTO_NPL dataset, the model has learned diverse biosynthetic transformations and has successfully predicted biosynthetic pathways for various natural products, such as broussochalcone A, leonurine and colibactin. AI also aids enzyme discovery for predicted reactions. For example, a neural network was trained on a dataset of the tissue‐specific transcriptome of *Atropa belladonna* , containing 43,861 transcripts across 11 different tissues. The neural network has successfully predicted tropane alkaloid transporters, addressing the bottleneck in the production of hyoscyamine and scopolamine, medicinal tropane alkaloids (Srinivasan and Smolke 2021). In another study, eight support vector machine models were developed to unveil missing enzymes (i.e., acetaldehyde synthase and phenylpyruvate decarboxylase) in the biosynthetic pathway of reticuline, a benzylisoquinoline alkaloid, in *Papaver somniferum* , demonstrating its usefulness for metabolic pathway reconstruction (Vavricka et al. 2022).

Although examinations focus on AI‐driven metabolic pathway reconstruction for the microbial production of therapeutic chemicals, all steps of metabolic engineering, including metabolic flux optimisation (Vaishnav et al. 2022; Seo et al. 2023) and fermentation (Huang et al. 2023), are being accelerated by the aid of AI (Kim et al. 2020). The synergy of AI and microbial technology promises the microbial production of various therapeutic chemicals, contributing to societal health and sustainability.

## 5 Concluding Remarks

The integration of AI with microbial technologies is revolutionising biomedical research, redefining our approach to infectious diseases, therapeutic interventions and antibiotic production. The synthesis of cutting‐edge technologies holds transformative potential for global health. In the context of infectious disease management, the crucial role of AI in early warnings, diagnosis and disease tracking with biomarkers provides dynamic insights into human health. Consequently, the analysis of microbial data by AI enhances global health informatics systems, especially in a rapidly evolving medical landscape. Microbial technologies, such as microbiome‐based therapeutics and phage therapy, have emerged as innovative strategies for disease prevention and treatment. They lay the foundation for practical use in clinical settings. Therefore, as more data accumulate and their applications expand, the application of AI could significantly increase the precision and impact of predictions in treatment. In the field of fundamental antibiotics and drugs, AI is used to design new types of drugs or medical substances that target specific microbes as pathogens. AI extends its influence to the production of therapeutic chemicals through metabolic engineering. This synergy promises a diversified portfolio of microbial production for therapeutic chemicals, contributing to health and sustainability. In conclusion, the collaborative efforts of AI and microbial technologies represent a paradigm shift in healthcare, offering unprecedented opportunities for innovation, efficiency and sustainability, shaping a future where precision defines the landscape of global health.

While microbial technologies enhanced by AI have led to significant advances for healthcare, several challenges need to be addressed to maximise their potential. In healthcare, even a modest rate of false positives or false negatives can lead to misdiagnosis or inappropriate treatment recommendations, implying the need for highly accurate models. Therefore, ensuring high‐quality datasets by addressing issues of noise and imbalance in datasets is essential as these factors could significantly undermine prediction performance. By developing advanced strategies for data curation, augmentation and rigorous validation, the field can pave the way for robust, high‐performing models that reliably inform healthcare applications. Furthermore, current AI interpretation approaches primarily identify correlations within microbial datasets, rather than clarifying the causal mechanisms underlying disease or medicinal effectiveness. This limitation hampers our understanding of disease processes and reduces confidence in AI‐driven predictions. Addressing these issues is essential to ensure that AI‐based approaches in microbial biotechnology can deliver reliable, precise and clinically relevant outcomes.

The integration of microbial technologies and AI not only shapes the trajectory of human–microbe interactions but also encourages a reconsideration of our relationship with the environment. The historical struggle against emerging microbes has highlighted the connections between public health challenges and environmental relationships. As prevention and treatment modalities changed during the COVID‐19 pandemic, advancements in AI triggered paradigm‐shifting ideas. Global sharing of information and big data utilisation has facilitated the translation of abstract trends in microbial data into practical models. Representatively, AI's role in unveiling the connection between human lifespan and changes in the gut microbiome due to dietary habits underscores its potential for influencing lifestyle choices (Zhang, et al. 2023b). Geographical and climate influence analyses driven by AI provide tools for proactive measures and public health alerts, demonstrating the technology's capacity to respond to external factors (Farooq et al. 2022). The ‘One Health’ concept, promoted by the WHOsation, advocates for a collaborative, multidisciplinary approach to achieve better public health outcomes. AI‐assisted microbial technology stands at the forefront, considering the fundamental microbial system and exploring the feasibility of a systematised approach. From essential functions in corners of the natural world to human health, changing microbiomes, whether they are adapted to nature or are manipulated, affect environmental implications. The potential of combining AI and microbial techniques is immense for understanding such complex interactive systems. Encouraging public and private partnerships, this approach emphasises a holistic understanding that goes beyond individual health, recognising the interconnectedness of human, microbial and environmental systems. This collaborative approach, driven by AI and microbial technologies, holds promise for addressing global health challenges in an integrated manner.

## Author Contributions

**Taeho Yu:** conceptualization, visualization, writing – original draft preparation, writing – review and editing. **Minjee Chae:** visualization, writing – original draft preparation, writing – review and editing. **Ziling Wang:** visualization, writing – original draft preparation, writing – review and editing. **Gahyeon Ryu:** writing – original draft preparation, writing – review and editing. **Gi Bae Kim:** writing – original draft preparation, writing – review and editing. **Sang Yup Lee:** conceptualization, funding acquisition, project administration, supervision, writing – original draft preparation, writing – review and editing.

## Conflicts of Interest

The authors declare no conflicts of interest.

## Data Availability

Data sharing is not applicable to this article as no new data were created or analyzed in this study.
